# A review of deubiquitinases and thier roles in tumorigenesis and development

**DOI:** 10.3389/fbioe.2023.1204472

**Published:** 2023-05-12

**Authors:** Xian-Wen Liang, Sheng-Zhong Wang, Bing Liu, Jia-Cheng Chen, Zhi Cao, Feng-Ran Chu, Xiong Lin, Hui Liu, Jin-Cai Wu

**Affiliations:** ^1^ Department of Hepatobiliary and Pancreatic Surgery, Hainan General Hospital (Hainan Affiliated Hospital of Hainan Medical University), Haikou, China; ^2^ Department of Gastrointestinal Surgery, Central South University Xiangya School of Medicine Affiliated Haikou Hospital, Haikou, China

**Keywords:** deubiquitinase, deubiquitination, tumor treatment, deubiquitinase inhibitor, cancer

## Abstract

Ubiquitin is a small protein that can be added onto target protein for inducing target degradation, thereby modulating the activity and stability of protein. Relatively, deubiquitinases (DUBs), a class catalase that can remove ubiquitin from substrate protein, provide a positive regulation of the protein amount at transcription level, post-translational modification, protein interaction, *etc.* The reversible and dynamic ubiquitination-deubiquitination process plays an essential role in maintaining protein homeostasis, which is critical to almost all the biological processes. Therefore, the metabolic dysregulation of deubiquitinases often lead to serious consequences, including the growth and metastasis of tumors. Accordingly, deubiquitinases can be served as key drug targets for the treatment of tumors. The small molecule inhibitors targeting deubiquitinases has become one of the hot spots of anti-tumor drug research areas. This review concentrated on the function and mechanism of deubiquitinase system in the proliferation, apoptosis, metastasis and autophagy of tumor cells. The research status of small molecule inhibitors of specific deubiquitinases in tumor treatment is introduced, aiming to provide reference for the development of clinical targeted drugs.

## 1 Introduction

The synthesis and degradation of organics are the essential processes for maintaining the homeostasis of biological metabolism. The Ubiquitin, a small protein (76-nt) extensively distributed within eukaryotes, can bind to substrate proteins through the ubiquitination process, and then induce its degradation *via* ubiquitin-proteasome system, which represents an important endogenous protein degradation pathway (Suresh et al., 2016). Ubiquitination, a critical proteins’ post-translational modification pathway, is mainly completed *via* three proteases. First, the ubiquitin-activating enzyme E1 initiates the ubiquitination process by activating ubiquitin in dependence on ATP. Then, the activated ubiquitin is transferred onto the ubiquitin-binding enzyme E2. Subsequently, the target protein is recognized with the cooperation of ubiquitin ligase E3. Therefore, ubiquitin can bind to the target protein’s Lys residue, thereby triggering a series of enzymatic reactions and eventually inducing hydrolysis of target protein by protease (Skaar et al., 2014). The ubiquitination process can be divided into three forms depending on the amount of the ubiquitin involved, namely, mono ubiquitination, multiubiquitination and polyubiquitination. Of them, mono ubiquitination only adds one individual ubiquitin onto the lysine residue of substrate protein, while multiubiquitination can add multiple ubiquitins onto several different lysine residues in one substrate. If multiple ubiquitin monomers link to each other through different Lys residues (Lys6, Lys11, Lys27, Lys29, Lys33, Lys48 and Lys63) or Met1 residues, the target protein can form a polyubiquitination chain (Komander and Rape, 2012).

**FIGURE 1:**
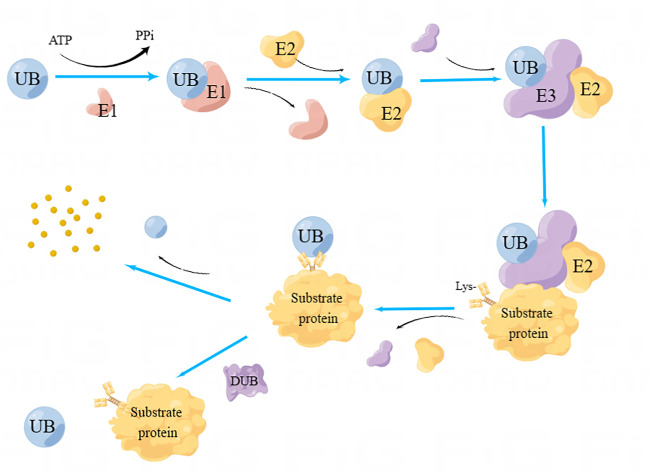
Process of Ubiquitination and deubiquitination. Ubiquitin-activating enzyme E1 initiates the ubiquitination process by activating ubiquitin in dependence on ATP. Subsequently, the activated ubiquitin is transferred onto the ubiquitin-binding enzyme E2. Subsequently, the target protein is recognized with the cooperation of ubiquitin ligase E3. As a result, ubiquitin can bind to the target protein’s Lys residue, thereby triggering a series of enzymatic reactions and eventually inducing hydrolysis of target protein by protease. DUBs can hydrolyze the peptide bond of ubiquitin, separate ubiquitin from the substrate protein, and induce deubiquitination.

Ubiquitination is a reversible dynamic process because deubiquitinases (DUBs) can hydrolyze the peptide bond of glycine at position 76 of ubiquitin, thereby removing ubiquitin from the substrate protein (Amerik and Hochstrasser, 2004). Deubiquitinase regulates protein function by mediating the deubiquitination of substrate proteins, and participates in various life activities of cells. During the process of tumor occurrence and development, there are many important tumor suppressor or cancer-promoting proteins associated with tumors that are regulated by deubiquitinase. The DNA damage repair, apoptosis, autophagy, and other cellular processes will be impacted by abnormal expression and mutation of deubiquitinase, which will regulate the growth, invasion, and metastasis of tumor cells. The dynamic balance between ubiquitination and deubiquitination maintains the healthy growth of cells. The ubiquitination and deubiquitination processes are shown in Figure 1.

Up to now, over 4 90 DUBs from seven subfamilies (Clague et al., 2019) have been discovered, including ubiquitin-specific proteases (USP), ubiquitin C-terminal hydrolases (UCH), ovarian tumor proteases (OTU), MachadoJosephin domain proteases (MJD), zinc-finger containing ubiquitin peptidase (ZUP1), Motif Interacting with Ub-containing novel DUB family (MINDY), as well as Jab1/Mov34/MPN + protease (JAMM) family includes zinc-binding metalloproteases (Suresh et al., 2016; Lange et al., 2022). DUBs-mediated deubiquitination has a complicated mechanism. DUBs can not only remove ubiquitin, but also promote the process of ubiquitin by recycling ubiquitination molecules, proofreading of ubiquitination, along with decomposition of ubiquitination inhibitors (Sowa et al., 2009). Deubiquitination is closely related to various cellular biological processes, and plays a vital role in the growth and metastasis of various tumors, including liver cancer, breast cancer (BC), colorectal cancer (CRC) and lung cancer, etc (Lange et al., 2022). At present, DUBs-targeting small-molecule inhibitors are becoming the hotspot anticancer therapeutic agents. Previous reviews on DUBs mainly introduced the molecular mechanism of one specific enzyme (Tao et al., 2022; Oikawa et al., 2023). This review aimed to introduce the effects of DUBs on tumor progression, and the impacts of DUBs on tumor cell growth, apoptosis, metastasis and autophagy, as well as the current research situation and development of DUBs inhibitors.

## 2 Effect of DUBs on tumor cell proliferation

A variety of DUBs have critical effects on the proliferation of different cancer cells (Liu et al., 2022). Typically, p53 is defined as a tumor suppressor. It can induce cell cycle arrest, apoptosis and senescence under the pressure of gene damage and carcinogenesis in order to maintain the integrity of the genome (Kastenhuber and Lowe, 2017; Uehara and Tanaka, 2018). In addition, it also can induce the proliferation, invasion, metastasis and immune escape of invasive tumor cell (Alexandrova et al., 2015; Bates, 2016; Cortez et al., 2016). USP7 can keep p53 expression level through deubiquitination (Tavana and Gu, 2017), thereby inhibiting the proliferation of tumor cells (Becker et al., 2008). In liver cancer cells, USP7 can be recruited by scaffold protein Abraxas brother 1 (ABRO1) to induce p53 deubiquitination. It has been confirmed to have the ability to inhibit the proliferation and colony formation of tumor cells *in vitro* (Zhang et al., 2014). USP7 also can deubiquitinate Mdm2, a p53 chaperone molecule and inhibitor, thus inhibiting its degradation (Cummins et al., 2004; Xia et al., 2021). In contrast, miR-205 that can inhibit the expression of USP7 can downregulate p53 and its downstream target protein, thereby promoting cell proliferation (Zhu et al., 2015).

USP21 can catalyze the deubiquitination of MEK2, thereby activating the ERK pathway, inducing cell proliferation and promoting tumor growth (Li et al., 2018). It also can deubiquitinize forkhead box M1 (FOXM1, a transcriptional factor), which plays an important role in the mitotic cycle of cells (Laoukili et al., 2005). In BC cells, loss of USP21 can arrest cell cycle and suppress cell growth (Peng et al., 2016), which are possibly through the regulation of FOXM1 (Arceci et al., 2019; Li et al., 2022).

Androgen receptor (AR) can suppress tumor cell proliferation through suppressing ERα signaling of ER-positive BC, thereby promoting ER-negative BC cell growth (Peters et al., 2009; Cochrane et al., 2014; Mina et al., 2017; Hou et al., 2021). USP14 can inhibit AR degradation by deubiquitination. Therefore, USP14 inhibitors suppress cell proliferation by causing the arrest of the cell cycle of AR-positive BC cells (Liao et al., 2018). Moreover, USP14 can activate phosphatidylinositol-3 kinase (PI3K) to induce cell growth *via* Wnt/β-catenin pathway (Huang et al., 2015; Zhang et al., 2020a), which also can contribute to the occurrence of drug resistance in CRC cells using USP22 (Miao et al., 2022). USP22 can deubiquitinize CCND1 protein and increase its expression level, thereby promoting the proliferation of CRC cells (Gennaro et al., 2018). In addition, it has also been shown that PSMD14 can promote cell growth *in vitro* and tumor development *in vivo* (Lv et al., 2020).

**FIGURE 2:**
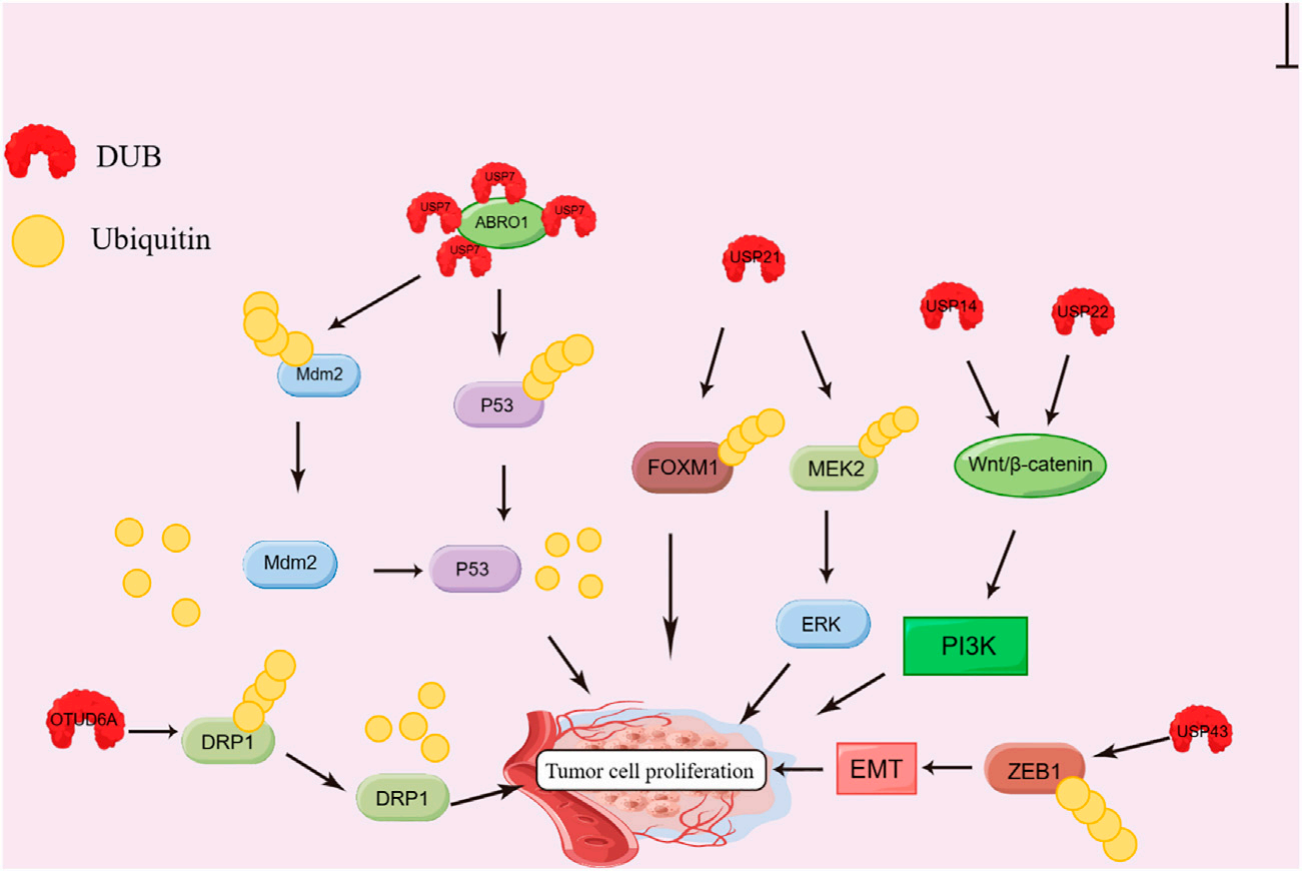
Effect of DUBs on tumor cell proliferation. ABRO1 recruits USP7 and induces deubiquitination of Mdm2 and P53; USP21 induces FOXM1 and MEK2 deubiquitination; USP14 and USP22 can activate PI3K *via* the Wnt/β-catenin pathway. USP43 inhibits the degradation of ZEB1 protein by deubiquitination; OTUD6A can stabilize DRP1 by deubiquitination; All the processes promote the proliferation of tumor cells.

The expression of USP14, USP21 and PSMD14 significantly increases within tumor tissue from liver cancer patients, which is negatively correlated with survival rate (Huang et al., 2015; Li et al., 2018; Lv et al., 2020). In prostate cancer, USP7, USP14, and USP22 can promote cell proliferation *via* AR, while USP16 inhibits cell proliferation and colony formation (Schrecengost et al., 2014; Liao et al., 2017; Ge et al., 2021). In addition, OTUD6A is reported to upregulate dynamin-related protein 1 (DRP1) by deubiquitination, thus promoting CRC cell proliferation (Shi et al., 2020). USP43 can enhance CRC cell growth by inducing the deubiquitination of Zinc finger E-box-binding homeobox 1 (ZEB1) protein, which is closely associated with tumor cell proliferation, invasion, migration and expression of EMT-related biomarkers. (Ye et al., 2021). In pancreatic cancer and non-small cell lung cancer (NSCLC), various DUBs have also been found to be highly expressed, including OTUD3, USP5, USP17, USP21, USP26, and USP28, which usually predicts dismal prognostic outcome (Lu et al., 2018; Wang et al., 2018; Du et al., 2019; Hou et al., 2019; Hu et al., 2019; Li et al., 2020a; Zhang et al., 2020b). The mechanism of DUB promoting tumor cell proliferation is shown in Figure 2.

## 3 Effect of DUBs on tumor cell apoptosis

MYC makes a vital effect on tumor cell growth and apoptosis. It can be classified into three types, c-MYC, N-MYC and L-MYC. Ubiquitination is a predominant pathway for MYC degradation (Chen et al., 2018a; Yoshida, 2018). Both USP28 and USP36 can bind to c-MYC in cells, and deubiquitinize c-MYC, thereby modulating cancer cell growth and apoptosis (Popov et al., 2007; Sun et al., 2015). USP13 can antagonize ubiquitin ligase FBXL14 mediated ubiquitination to stabilize c-MYC, finally maintaining tumorigenicity and self-renewal of germline stem cells (Fang et al., 2017). Moreover, USP13 can deubiquitinize myeloid-cell leukemia 1 (MCL1, an anti-apoptotic protein). Therefore, if USP13 inhibition reduces the protein level of MCL1, it significantly elevates cancer cell apoptosis. Pannem et al. discovered CYLD expression was negatively related to the expression levels of proliferation markers Ki-67 and c-MYC, as evidenced by tissue microarray and immunohistochemistry (IHC) analysis of human liver cancer samples. On the contrary, CYLD silencing can induce tumor cell growth (Pannem et al., 2014).

For AR-positive BC cells, USP14 can deubiquitinize the anti-apoptosis effect of AR, and inhibiting USP14 can induce the cleavage of polyadenosine diphosphate ribose polymerase 1 and reduce BCL2 protein level, thereby promoting apoptosis (Liao et al., 2018). However, PSMD14 shows the opposite effect (Luo et al., 2017). In CRC cells, various DUBs are tightly associated with apoptotic proteins. For instance, USP22 can regulate cell apoptosis by regulating mTOR (Kosinsky et al., 2020), while USP1 can inhibit the degradation of myeloid-cell leukemia 1 (MCL1) and B-cell lymphoma 2 (BCL2), both of which are known as anti-apoptotic proteins (Xu et al., 2019). Other studies have found mutations of USP17 or OTUD1 gene within pancreatic cancer cells. This leads to expression of yes-associated protein 1 (YAP) and the decrease of nuclear factor erythroid 2-related factor 2 (NRF2), eventually regulating cell apoptosis (Chen et al., 2020; Grattarola et al., 2021). DUBs related to DNA damage repair have been reported to be associated with apoptosis in NSCLC tissues. For example, USP35 can reduce the endoplasmic reticulum stress-induced apoptosis by stabilizing ribosomal binding protein 1 (RRBP1) (Garcia-Santisteban et al., 2013; Wang et al., 2022). UCHL1 and WSP9X can regulate cell apoptosis by inhibiting p53 degradation in HCC tissues (Yu et al., 2008; Liu et al., 2015).

## 4 Effect of DUBs on tumor cell metastasis

Metastasis, a typical characteristic of cancer that causes high mortality, can also be affected by DUBs. For example, OTUB1 can promote the progression, metastasis and invasion of gastric cancer (GC), prostate cancer and colon cancer (Zhou et al., 2014; Iglesias-Gato et al., 2015; Weng et al., 2016; Zhou et al., 2018). Relatively, OTUD1 also can inhibit tumor metastasis by preventing the degradation of SMAD family member 7 (SMDA7), which can inhibit tumor metastasis by blocking TGF-β signaling pathway (Zhang et al., 2017). In addition, OTUB3 exhibits different biological functions in various tumors. In BC tissues, OTUD3 knockout can activate Akt pathway, inducing cell transformation and tumor metastasis (Yuan et al., 2015). However, in lung cancer tissues, its expression is upregulated, which predicts dismal prognostic outcome (Du et al., 2019; Zhang et al., 2020a). The underlying mechanism is that OTUD3 induces the deubiquitination of glucose-regulated protein 78 (GRP78), and subsequently promotes tumor proliferation and metastasis (Qian et al., 2017; Shen et al., 2017).

Many ubiquitin-specific protease family members are also connected with tumor metastasis. USP4 is a vital cell pathway regulator, which is involved in p53 regulation, TGF-β response and NF-κB signal transduction, and makes a critical effect on cancer genesis and progression (Clerici et al., 2014; Qiu et al., 2018; Zhang et al., 2020b). In CRC tissues, USP4 has been found to stabilize PRL-3 by deubiquitinating PRL-3, activating PI3K/AKT pathway and down-regulating E-cadherin, thereby promoting tumor proliferation and metastasis (Xing et al., 2016). In lung cancer tissues, USP4 can stabilize Twist1 to promote tumor cell stemness (Li et al., 2020b). In addition, USP9X can regulate EMT, while USP37 can stabilize c-MYC by the deubiquitination of USP21 and YY1, aiming to promote the metastasis of lung cancer cells (Pan et al., 2015; Cai et al., 2019; Xu et al., 2020). In conclusion, DUBs are closely related to the metastasis of a variety of tumors, and the treatment of targeted DUBs is expected to inhibit tumor metastasis.

## 5 Effect of DUBs on tumor autophagy

In eukaryotes, autophagy is a highly conserved lysosomal degradation pathway that recognizes and degrades dysfunctional organelles, macromolecular complexes, as well as intracellular bacteria and other foreign bodies. Moreover, it generates energy recycling, and makes a critical effect on maintaining homeostasis of cells, tissues, and organisms (Li et al., 2020c). Autophagy has been found to be related to many disorders including microbial infection, Alzheimer’s disease (AD), cancer progression and drug resistance (Qiu et al., 2022). Nevertheless, the relation between autophagy and tumors is extremely complicated and controversial. Autophagy can protect tumor cells through recycling degraded products and removing injured organelles. However, excess autophagy may paradoxically cause “type II programmed cell death” or “autophagic cell death” in cancer cells (Chen et al., 2018b; Qiu et al., 2022). Autophagy resulted from drug treatment and metabolism has shown a dual-effect on cancer resistance. In a word, it not only causes apoptosis but also promotes cell survival (Yu et al., 2019). Therefore, how to use autophagy appropriately in cancer drug therapy is of particular importance.

Ubiquitination, as an important post-translational modification, participates in several autophagy stages (Grumati and Dikic, 2018). Accordingly, DUBs also have key regulatory effects on autophagy by targeting the ubiquitinated autophagic regulatory components or autophagic substrates (Magraoui et al., 2015).

Mammalian target of rapamycin complex 1 (mTORC1) and ULK1/2 kinase complex are key complexes that induce autophagy. DEPTOR is a mTORC1 inhibitor that can regulate autophagy (Zhao et al., 2011). OTUB1 can directly bind to DEPTOR through the N-terminal domain, which can thereby enable the deubiquitination of DEPTOR, increasing the DEPTOR expression level and inhibiting the activity of mTORC1 (Zhao et al., 2018). USP1, USP20 and USP24 can interact with ULK1 to catalyze its deubiquitination and protect it against degradation, eventually inducing autophagy (Nazio et al., 2016; Kim et al., 2018; Raimondi et al., 2019; Thayer et al., 2020). USP30 and USP33, which are localized in the mitochondrial outer membrane, can interact with the E3 ubiquitin ligase Parkin, thereby negatively regulating mitophagy (Bingol et al., 2014; Michel et al., 2017; Niu et al., 2020; Zhang et al., 2022a). In addition, USP15 and USP35 have also been found to play a negative regulation role in mitophagy (Cornelissen et al., 2014; Wang et al., 2015). By contrast, USP8 can positively regulate mitophagy through specifically eliminating K6 ubiquitin chain on Parkin. Silencing USP8 can prevent the recruitment of Parkin into the depolarized mitochondria, thereby inhibiting Parkin-mediated mitophagy (Durcan et al., 2014; Durcan and Fon, 2015). The targeted proteins and regulatory mechanisms of DUBs are presented in Table 1.

**TABLE 1:** DUBs involved in autophagy.

DUB	Target proteins	Regulatory mechanisms	Effects on autophagy of tumor cells
OTUB1	DEPTOR Zhao et al., 2018	It mediates DEPTOR deubiquitination and inhibits mTORC1 activity Zhao et al., 2018	Promote
USP1	ULK1 Raimondi et al., 2019	USP1 hydrolyzes the K63 ubiquitin chain of ULK1 and regulates the dynamic activity of ULK1 autophagy initiation and postinitiation Raimondi et al., 2019	Promote
USP20	. ULK1 Kim et al., 2018	USP20 hydrolyzes the K48 ubiquitin chain of ULK1 Kim et al., 2018	Promote
USP14	TAB 2 Min et al., 2017	Inhibiting TrAF6-mediated Beclin-1 K63 ubiquitination Min et al., 2017	Inhibit
USP19	Beclin1 Jin et al., 2016	Hydrolyzing the K11 ubiquitin chain at Lys 437 to protect Beclin-1 from degradation Jin et al., 2016	Promote
USP33	RALB Simicek et al., 2013	Indirectly regulating Beclin-1 by deubiquitination of RALB Simicek et al., 2013	Inhibit
	Parkin Niu et al., 2020	Hydrolyzing the K63 ubiquitin chain at Lys435 of Parkin protein Zhou et al., 2018	
USP8	EPG5 Gu et al., 2019	Promoting the fusion of autophagosome and lysosome, and hydrolyzing the K63 ubiquitin chain at EPG5 Lys 252 Gu et al., 2019	Promote
	Parkin Durcan and Fon, (2015)	Hydrolyzing the K6 ubiquitin chain on Parkin Durcan and Fon, (2015)	
USP35/USP15	mitochondrial protein [ Wang et al., 2015, Cornelissen et al., 2014	Reverse ubiquitination of Parkin substrates on mitochondria Wang et al., 2015, Cornelissen et al., 2014	Inhibit

## 6 Small-molecule inhibitors of DUBs

Due to the extensive involvement of DUBs in tumour proliferation, apoptosis and metastasis, numerous small molecule inhibitors of DUBs have been identified as tumor drugs. At present, most inhibitors of DUBs are still in the research stage. VLX1570, a USP14/UCHL5 dual inhibitor, was the first DUB inhibitor investigated clinically in 2015. However, its investigation was terminated in the clinical trial stage due to its dose-limiting toxic effect (Wang et al., 2016; Schauer et al., 2020). Most of these DUBs inhibitors for cancer therapy are targeting USP family members, and non-USP family inhibitors need to be further investigated. P22077 can selectively downregulate USP7, USP10 and USP47, thereby effectively inhibiting the cell proliferation of neuroblastoma and T-cell acute lymphoblastic leukemia (ALL), and inducing apoptosis (Fan et al., 2013; Shan et al., 2018; Gu et al., 2022). In addition, it can suppress the metastasis of melanoma cells (Vishnoi et al., 2018). B-AP15 contributes to the selective inhibition of USP4 and UCHL5, and restrains the cell proliferation of various cancers, including lung cancer, CRC, BC, myeloma, prostate cancer, and liver cancer (D'Arcy et al., 2011; Tian et al., 2014; Cai et al., 2017; Ding et al., 2018). As the s, P5091 is also an elective dual-inhibitor of USP7 and USP47 that can suppress CRC cell growth and induces their apoptosis *in vitro* (An et al., 2017). In addition, it can hinder the proliferation of ovarian cancer cells and promote the apoptosis. The effect is more effective on ovarian cancer than wild-type p53 (Wang et al., 2017a; An et al., 2017).

Focusing on autophagy that makes a critical effect on tumor development, researchers have successively identified and screened various specific small-molecule inhibitors of autophagy-related DUBs, including USP1-UAF1 complex inhibitors GW7647 and Pimozide, which can successfully reverse NSCLC cell resistance to chemotherapeutic drugs (Chen et al., 2011). Small-molecule inhibitor Spautin-1 can increase the ubiquitination level of Beclin1 subunit in VPS34-PI3K kinase complex and promote its degradation by suppressing the activities of USP10 and USP13, thus preventing the process of cell autophagy. The combination of DUBs inhibitors and anticancer drugs can enhance the sensitivity of tumor cells to drugs (Shao et al., 2014; Schott et al., 2018; Yeo et al., 2018; Dong et al., 2019). The treatment of H1299 lung cancer cells with LDN-57444, a small-molecule inhibitor targeting UCH-L1, significantly decreased the proliferation rate of tumor cells (Liu et al., 2003). WP1130 can partially selectively inhibit DUBs, which can directly inhibit deubiquitin activities of USP5, USP9X, USP14, UCH37, and UCH-L1, resist the proliferation and promote the apoptosis of various tumor cells (Wang et al., 2017b; Kim et al., 2019). Deubiquitinase inhibitors were shown in Table 2.

**TABLE 2:** Deubiquitinase inhibitors in tumor therapy.

Inhibitor	Target	Disease
VLX1570	USP14/UCHL5 [ Durcan and Fon, (2015), Durcan et al., 2014	Multiple myeloma Durcan et al., 2014
P22077	USP7, USP10, USP47 Wang et al., 2016; Schauer et al., 2020; Gu et al., 2022	Neuroblastoma Wang et al., 2016, T-cell acute lymphoblastic leukemia Gu et al., 2022
B-AP15	USP 4, UCHL5 Shan et al., 2018; Vishnoi et al., 2018; D'Arcy et al., 2011; Tian et al., 2014	Lung cancer Shan et al., 2018, multiple myeloma Vishnoi et al., 2018, prostate cancers D'Arcy et al., 2011, hepatocellular carcinoma Tian et al., 2014
P5091	USP7, USP47 An et al., 2017; Cai et al., 2017; Ding et al., 2018	Colorectal cancer Cai et al., 2017 and Ovarian Cance r Ding et al., 2018, An et al., 2017
Pimozide, GW7647	USP1-UAF1 Wang et al., 2017a	non-small cell lung cancer Wang et al., 2017a
Spautin-1	USP10, USP13 Chen et al., 2011; Shao et al., 2014; An et al., 2017; Schott et al., 2018	chronic myeloid leukemia An et al., 2017, osteosarcoma Chen et al., 2011
		breast cancer Shao et al., 2014
LDN-57444	UCH-L1 Yeo et al., 2018	Lung cancer Yeo et al., 2018
WP1130	USP5, USP9X, USP14, UCH37, UCH-L1 Liu et al., 2003; Dong et al., 2019	Renal carcinoma Dong et al., 2019, Lung cancer Dong et al., 2019, hepatocellular cancer Dong et al., 2019, and multiple myeloma Liu et al., 2003

## 7 Conclusion

Over the past decades, the structure, function, role, associated mechanism, and the relationship with diseases of DUBs have been investigated. It has been known that DUBs are closely associated with cancer genesis and progression, which have become the new hotspots in tumor treatment. DUBs are extensively involved in various processes of tumor development. They can regulate the levels of proteins, including the “undrugable” targets that are not sensitive to traditional targeted therapies. Therefore, DUBs have a broad prospect as the tumor therapeutic targets. However, most of the DUBs have poor specificity. One DUB can regulate multiple substrates, while one substrate also can be regulated by multiple DUBs. In addition, there are dynamic changes between ubiquitin ligases and DUBs. Therefore, in various types of tumors, DUBs show a dual role of promotion and suppression. The original methods and techniques for screening inhibitors are also associated with some shortcomings such as poor biocompatibility and high off-target rate (Harrigan et al., 2018), causing difficulties in the clinical translation of DUBs inhibitors. With significant improvements in biochemical analysis and screening techniques, an increasing number of small-molecule DUBs inhibitors have been discovered. Transforming small-molecule inhibitors into specific probes to explore the downstream regulatory mechanisms of DUBs can provide a certain foundation for the clinical evaluation of drug-like molecules, and also provide various tools for further analysis of related regulatory processes, biochemical mechanisms, and drug efficacy evaluation in disease models. We believe that DUBs and their inhibitors have a bright future in the field of cancer therapy, and more exciting results will emerge in the future, promoting them to eventually become the reliable clinical targets for cancer therapy.
